# The Relationship Between Physical Activity and Gait Rhythm with Motor Imagery -Trial Using the Finger Tap Test-

**DOI:** 10.3390/jfmk10010094

**Published:** 2025-03-17

**Authors:** Keisuke Itotani, Mirai Taki, Shinnosuke Ueno, Hina Nakai, Yuta Miki, Ippei Suganuma, Shun Harada, Noriyuki Ogawa

**Affiliations:** 1 Department of General Rehabilitation, Faculty of Allied Health Sciences, Yamato University, 2-5-1 Katayama-cho, Suita 564-0082, Japan; 2 Department of Physical Therapy, Faculty of Therapy, Tokuyukai Medical Corporation, Kansai Rehabilitation Hospital, 3-11-1 Sakurano-cho, Toyonaka City 560-0054, Japan; 3 Department of Physical Therapy, Faculty of Therapy, Tokusyukai Medical Corporation, Suita Tokusyuukai Hospital, Senrioka-nishi 21-1, Suita 565-0814, Japan; 4 Department of Physical Therapy, Faculty of Therapy, Seifukai Medical Corporation, Hanshin Rehabilitation Hospital, 1-59-3 Ono, Itami City 664-0003, Japan; 5 Department of Physical Therapy, Faculty of Therapy, Wafukai Medical Corporation, Senri Rehabilitation Hospital, 4-6-1 Onohara-west, Mino City 562-0032, Japan; 6 Department of Occupational Therapy, Faculty of Health Sciences, Kyoto Tachibana University, 34 Oyakeyamada-cho, Yamashina-ku, Kyoto 607-8175, Japan; suganuma-i@tachibana-u.ac.jp (I.S.); harada-s@tachibana-u.ac.jp (S.H.); ogawa-n@tachibana-u.ac.jp (N.O.)

**Keywords:** beats per minute, delta rhythm, maximum walking, mental imagery, motor imagery

## Abstract

Objectives: The purpose of this study was to investigate the relationship of any error (delta; ∆) between the image of one’s own walking rhythm and the actual walking rhythm and physical activity, as a new motor imagery assessment. Methods: The subjects were classified into two groups: a high activity group (HA-Group) having high physical activity with less than four hours of sitting time per day, and a low activity group (LA-Group) having low physical activity with more than four hours of daily sitting time. Visual rhythm, auditory rhythm, mental comfortable walking rhythm, and mental maximum walking rhythm were used to assess new motor imagery. Their beats per minute were measured and any error (delta; ∆) from the actual rhythm was calculated: ∆ visual rhythm, ∆ auditory rhythm, ∆ mental normal gait rhythm, and ∆ mental maximal gait rhythm. Results: When comparing the two groups, the HA-Group had significantly higher ∆ visual rhythm, lower ∆ auditory rhythm, higher ∆ mental comfortable walking rhythm, and lower ∆ mental maximum walking rhythm ability than the LA-Group. Furthermore, in an ANCOVA with age, ∆visual rhythm, and ∆auditory rhythm as adjustment factors, the HA-Group had significantly lower ∆mental maximum walking rhythm than the LA-Group. Conclusions: These results showed that the rhythmic assessment of the imagery of maximum walking was associated with stationery time. It is possible that the more inaccurate the imagery of maximum walking, the longer the sitting or lying time.

## 1. Introduction

Humans can imagine and manipulate various phenomena in their minds. This function is supported by mental representations. In particular, the mental representation of a movement that does not involve physical movement is defined as “motor imagery” [1]. When humans perform an exercise, an image of that exercise is unconsciously generated in advance [2,3]. In addition, consciously performing the motor image has the same mechanism as when the motor image is generated unconsciously, and they are reported to be functionally equivalent [2,3]. To date, various approaches to humans using motor imagery have been used: mental training using exercise imagery and investigation of muscle fatigue and muscle activity [4], substitution of exercise therapy when physical movement is restricted due to neurological disorders [5], approaches to flexibility and muscle strength using motor imagery training [6,7], and evaluating the accuracy of motor imagery by using the error between the estimated motor imagery and actual movement [8,9]. Furthermore, there is a report that motor imagery improves physical performance [10]. This study [10] investigated the effects of training using motor imagery on strength and power performance in professional athletes. The motor imagery training group performed resistance training exercises through mental rehearsal, while the control group underwent regular training (both groups trained for 6 weeks). The results indicated that the group using motor imagery for training showed improvements in lower limb muscle output and jumping ability compared to the control group.

However, on the other hand, it was also reported that the effect is smaller than actual performance training, so it is more effective to use motor imagery in combination with actual performance training [11]. Furthermore, mental fatigue training for movements that cannot be performed at all may be ineffective [12], and repeated mental fatigue may occur [13]. From these results, it is estimated that being able to imagine accurately is less likely to cause fatigue and is more likely to benefit the mind and body.

Motor imagery is predicted by applying one’s own current situation and state, and one must be able to closely monitor these factors. If the accuracy of the motor image and the actual movement is poor and the error is large, the movement will not be performed as predicted, which will interfere with the time schedule and physical performance. Previous studies [9,14,15] investigated this error using “delta; ∆” calculated from an actual Timed Up and Go test (TUG) and an imagined Timed Up and Go test (iTUG) based on motor imagery. If the value of “∆” is large, it means that the action oneself is about to perform cannot be properly predicted. Previous research [14] reported that this delta time is associated with falls. Furthermore, it was also reported that older adults, whose walking speed during dual tasks is slower compared to their normal walking speed, have a larger difference between their actual TUG and iTUG [16]. Therefore, evaluation of this error is attracting attention as a tool for early detection of cognitive impairment [17].

On the other hand, we discovered that this “∆” may occur not only in the older adults and stroke patients, but also in young people [9]. In that report [9], groups were classified according to the presence or absence of activity restriction, and when compared, no significant difference was observed between the groups in exercise performance, but a significant difference was observed only in ∆TUG. This means that when physical activity is restricted, that is, when the amount of physical activity decreases, predictions based on motor imagery may not be performed appropriately, regardless of age or disability. However, there was some lack of reliability as physical activity levels were not investigated and motor imagery was only evaluated using iTUG.

Based on these findings, it seemed necessary to newly investigate the relationship between physical activity and motor imagery. In physical activity, the number of steps was shown to be related to energy expenditure during physical activity [18]. Therefore, it seems that the higher the amount of physical activity, the more opportunities to walk, and the higher the amount of physical activity, the easier it is to imagine one’s own walking rhythm.

Therefore, in this study, we decided to investigate the relationship between actual physical activity and imagined physical activity, focusing on any discrepancy (error) between the image of one’s walking rhythm and the actual walking rhythm, in addition to iTUG, as a new evaluation of motor imagery. As a hypothesis of the study results, we assumed that individuals with higher levels of physical activity might exhibit errors between their imagined walking rhythm and actual walking rhythm compared to those with lower levels of physical activity.

## 2. Materials and Methods

### 2.1. Participants

A self-administered questionnaire survey and an evaluation of motor imagery and cognitive function were conducted for 15 community-dwelling older adults (age; 73.3 ± 5.6, height; 154.9 ± 7.7, weight; 52.4 ± 5.6) and 15 healthy young people (age; 20.3 ± 0.5, height; 162.1 ± 6.7, weight; 52.9 ± 7.4) who participated in a health measurement session held at a Suita City civic center. The exclusion criteria for the analysis were (1) those who could not provide consent, (2) those who did not complete all measurements, or (3) those who had at least one analytical datum missing. The study plan was provided in writing to all subjects before beginning measurements, and all subjects provided informed consent. This study was carried out after written consent was obtained from the subject. Furthermore, this study was approved by the Yamato University Faculty of Health Sciences Research Ethics Committee (approval number: R4013).

### 2.2. Materials

Assessment and measurement variables were age, height, weight, sex, daily activity time, sleep time, cognitive function, rhythmic ability, and motor imagery. The overall characteristics of the subjects obtained are shown in Table 1. Age, sex, daily activity time, and sleep time were investigated using a questionnaire. In addition, daily activity time was investigated using the IPAQ [19,20] “time spent sitting or lying down (time lying down)”. Sleep time was calculated by asking bedtime and wake-up time.

Cognitive function was assessed using the Japanese version of the Montreal Cognitive Assessment (MoCA-J). MoCA-J is used for trail making, 3D figure imitation, clock drawing, naming, attention (forward/reverse/target detection/calculation), language (sentence repetition/word recall), abstract thinking, delayed playback, and register. It consists of eight items of knowledge. Each item is judged as correct or incorrect and scored on a 30-point scale. A score of 26 or above is within the normal range, and a score of 25 or below is effective for the screening of mild cognitive impairment [21].

### 2.3. Rhythmic Ability Assessments

Rhythm ability was assessed using both visual and auditory modalities. Rhythm ability using visual modality was defined as visual rhythm, and rhythm ability using auditory modality was defined as auditory rhythm. For visual rhythm, the measurer was first asked to watch a video of a person walking, taken from the sagittal plane (the walking played in the video was pre-set to 100 beats per minute (BPM) and the subjects did not know this information). After watching the video, the measurer said, “Please tap the desk with your index finger at the timing when the left and right heels of the person walking in the video touch the ground.” While watching the same video again, subjects practiced tapping for 1 min. After practice, the subjects were asked to tap on the screen of a tablet placed on their desk while watching a walking video. Tap rhythm measurements were carried out using a free BPM heart rate measurement counter application When this application is launched, a “tap” message will be displayed on the tablet screen (a tap will automatically start the measurement process). Once measurement starts, the reset button, number of taps, time between taps, and BPM values are displayed in real time. In the measurement, the total number of taps by each subject was 30, and was counted by the measurer. The subject began tapping at the measurer’s “start” signal. Rhythm was measured not from the first tap, but from the sixth tap. Therefore, the measurer tapped the reset button immediately after the subject tapped five times. This reset button was tapped without it being noticed by the subject. The BPM of the taps was calculated for a total of 25 taps, from the 6th tap to the 30th tap. The obtained BPM value was entered to the first decimal place. Auditory rhythm was determined using Ladytron’s international dateline (168 BPM) music from a previous study [22]. The music was listened to twice. The first time, the subjects listened while standing still, and from the second time onwards, they tapped in time with the rhythm of the music, using the same method as the visual rhythm, and the BPM was measured. After the measurement, subjects were asked if they had ever listened to this music.

### 2.4. Motor Imagery Assessments

Motor imagery was assessed using the Timed Up and Go test (TUG), imaged Timed Up and Go test (iTUG) [9,14,15], and mental walking rhythm. The TUG and iTUG were measured as follows, based on previous studies [9,15]. For the TUG, the time was measured using a stopwatch as the participant stood up from a chair with a backrest and a seat height of about 45 cm, walked around a cone 3 m away, and sat back down. The measurement started when the participant sat back down and continued until they reached a comfortable walking speed. The measurement was taken from the signal of “Ready, Go” to the subject sitting back down in the chair, and at a comfortable walking speed. The iTUG was measured using a stopwatch following previous studies [19,20], and this measurement was always performed before TUG. First, the participant sat in the chair and imagined performing TUG. In other words, they visualized standing up from the chair, walking around a cone 3 m away, and sitting back down. The participant could choose whether to perform iTUG with their eyes open or closed. The stopwatch measurement started with the examiner’s command of “Ready, Go”, and stopped when the participant said “Stop”. The image of standing up from the chair was triggered by the examiner’s “Ready, Go”. The participant was instructed to shout “Stop” when imagining sitting down again, doing so loudly. The participant was asked to perform both iTUG and TUG at their usual speed. iTUG was performed once, followed by TUG, which was also performed once. Both times were recorded with a stopwatch to the nearest 0.01 s.

Mental walking rhythm was measured in two patterns: comfortable walking (C-walking) and maximum walking (M-walking). First, subjects were asked to imagine their own C-walking. Afterwards, subjects were asked to tap their fingers at the timing when both heels would touch the ground as they imagined walking. After practicing tapping according to their own walking image for 1 min, we began measuring their mental C-walking rhythm. BPM was measured in the same way as visual and auditory rhythm to measure the mental C-walking rhythm. After measuring the mental C-walking rhythm, the mental M-walking rhythm was also measured in the same way. Afterwards, actual C-walking and M-walking were recorded with a video camera, and BPM was measured from the actual C-walking rhythm and actual M-walking rhythm.

### 2.5. Data Analysis

Visual rhythm, auditory rhythm, iTUG, mental C-walking rhythm, and mental M-walking rhythm errors (delta; ∆) were calculated by comparing the actual and imagined values. For example, the error for TUG (∆TUG) was calculated using the formula: ∆TUG = [(TUG − iTUG)/(TUG + iTUG)/2)] × 100

Similarly, the errors for visual rhythm, auditory rhythm, mental C-walking rhythm, and mental M-walking rhythm were calculated by comparing the actual values with the corresponding imagined values, following the same formula, adjusting the reference values accordingly (100 for visual rhythm, 168 for auditory rhythm, and the actual walking rhythms for mental C-walking and M-walking).

Based on the results obtained from the IPAQ [19,20] for “time spent sitting or lying down,” we determined whether or not subjects spent more or less than 4 h per day of physically inactive time, which was shown as a borderline time for increasing the risk of death [23]. Subjects were then divided into two groups: a high activity group (HA-Group) of less than 4 h of daily inactivity, and a low activity group (LA-Group) of more than 4 h. Assessment and measurement variables were analyzed using Student’s *t* test and chi-square test. Additionally, comparisons were made using analysis of covariance (ANCOVA) adjusted for variables for which significant differences were observed in the Student’s *t*-test and chi-square test. The significance level was set at *p* < 0.05. Analysis was performed using IBM SPSS version 26 (IBM Corporation, Armonk, NY, USA).

## 3. Results

Due to missing data from 2 of the 30 subjects, the analysis was conducted on 28 participants. A total of 8 participants belonged to the HA-Group, and 20 to the LA-Group.

### 3.1. Group Attributes

Regarding the attributes of the two groups, the HA-Group was significantly older than the LA-Group, with a mean age of 65.1 ± 18.9 years for the HA-Group and 36.6 ± 25.8 years for the LA-Group (*p* = 0.009, r = 0.48). However, no significant differences were observed in height, weight, BMI, sex, or sleep time between the two groups, as shown in Table 1.

### 3.2. Cognitive Function (MoCA-J)

The MoCA-J assessment revealed that the HA-Group had significantly lower scores compared to the LA-Group. The mean score for the HA-Group was 24.1 ± 3.2, while the LA-Group scored 27.5 ± 2.2 (*p* = 0.004, r = 0.53).

### 3.3. Rhythm Variables

In the rhythm variables, significant differences were observed between the two groups. For visual rhythm, the HA-Group scored a mean of 98.3 ± 2.2, whereas the LA-Group scored 100.4 ± 2.7 (*p* = 0.049, r = 0.35). Similarly, auditory rhythm was higher in the HA-Group (178.4 ± 48.2) compared to the LA-Group (130.7 ± 53.9, *p* = 0.023, r = 0.41). The mental C-walking rhythm was also significantly different, with the HA-Group scoring 104.3 ± 24.0 compared to 82.3 ± 19.0 in the LA-Group (*p* = 0.017, r = 0.45). In the mental M-walking rhythm, the HA-Group scored 156.7 ± 42.0, which was significantly higher than the LA-Group’s score of 111.6 ± 27.5 (*p* = 0.002, r = 0.55).

### 3.4. Delta (∆) Variables

For the ∆ variables, significant differences were also observed. The mean ∆visual rhythm was 1.8 ± 2.2 in the HA-Group and −0.4 ± 2.6 in the LA-Group (*p* = 0.049, r = 0.35). In ∆ auditory rhythm, the HA-Group scored −2.2 ± 31.5, while the LA-Group scored 31.4 ± 38.5 (*p* = 0.023, r = 0.41). The ∆mental C-walking rhythm showed a mean of −37.6 ± 28.8 in the HA-Group and −5.8 ± 30.3 in the LA-Group (*p* = 0.015, r = 0.46). Finally, the ∆ mental M-walking rhythm was −8.9 ± 23.9 in the HA-Group and 35.9 ± 26.2 in the LA-Group (*p* = 0.001, r = 0.63).

### 3.5. Actual Walking Rhythm

In the actual walking rhythm, a significant difference was observed only in the M-walking rhythm. The HA-Group scored 139.1 ± 8.6, while the LA-Group scored 158.6 ± 24.1 (*p* = 0.025, r = 0.39). No significant differences were observed in other rhythm items, as shown in Table 2.

### 3.6. ANCOVA

ANCOVA with age as an adjustment factor revealed significant differences in the mental M-walking rhythm and ∆mental M-walking rhythm. The mental M-walking rhythm showed a *p* value of 0.031 (r = 0.17), while the ∆ mental M-walking rhythm showed a *p* value of 0.009 (r = 0.24), as presented in Table 3. When ANCOVA was conducted using age, ∆visual rhythm, and ∆auditory rhythm as adjustment factors, similar results were obtained. The mental M-walking rhythm showed a *p* value of 0.019 (r = 0.24), and the ∆mental M-walking rhythm showed a *p* value of 0.008 (r = 0.30), as presented in Table 4.

## 4. Discussion

This study aimed to investigate the relationship between physical activity and the error (∆) between one’s own walking rhythm image and the actual walking rhythm, as a new way to evaluate motor imagery. Therefore, physical activity was evaluated using the IPAQ [19,20] sitting or lying down time, based on previous research [23]. The subjects were classified into two groups: an HA-Group of high physical activity with sitting time of less than 4 h per day, and an LA-Group with 4 h or more of low physical activity levels. New motor imagery was evaluated using visual rhythm, auditory rhythm, mental C-walking rhythm, mental M-walking rhythm, ∆ visual rhythm, ∆ auditory rhythm, ∆ mental C-walking rhythm, and ∆ mental M-walking rhythm. Also, conventional motor imagery evaluations such as iTUG and ∆ TUG [12,22] were included, and analysis was performed using ANCOVA and a comparison between the HA-Group and LA-Group. In a comparison between the two groups, the HA-Group was significantly older than the LA-Group, but no significant differences were observed in height, weight, BMI, sex, and sleep time. In addition, the HA-Group had significantly lower MoCA-J values than the LA-Group, lower values for visual rhythm, higher values for auditory rhythm, higher values for mental C-walking rhythm and mental M-walking rhythm, and higher values for actual mental walking rhythm. M-walking rhythm was low, ∆ visual rhythm was high, ∆ auditory rhythm was low, ∆ mental C-walking rhythm was high, and ∆ mental M-walking rhythm was low. In ANCOVA with age as an adjustment factor, the HA-Group had significantly higher mental M-walking rhythm and lower ∆ mental M-walking rhythm than the LA-Group. Furthermore, an ANCOVA in which age, ∆visual rhythm, and ∆auditory rhythm were used as adjustment factors yielded similar results to the ANCOVA in which only age was used as an adjustment factor. These results showed that the rhythm evaluation when imagining M-walking was related to the time spent in a sitting or lying position.

A previous study has shown that iTUG and ∆TUG, which are evaluations of motor imagery, are influenced by the amount of physical activity [9]. However, in this study, no significant difference was observed between the HA-Group and LA-Group for iTUG and ∆TUG. This may have been strongly influenced by the fact that the subjects in the previous study [9] were forced to restrict their activities to non-essential outings and were extremely physically inactive compared to their normal daily life. Such a situation would probably have a negative impact on iTUG. However, the subjects in this study did not have any restrictions that forced them to limit their daily physical activity. Therefore, it seems that classifying physical activity by time spent sitting or lying down had no effect on the iTUG or ∆TUG. Therefore, when evaluating using motor imagery, it is important to selectively use the evaluation method depending on the subject’s physical activity status, or to prepare a variety of evaluation methods.

Although there was a difference in daytime sitting and lying time between the two groups in this study, no significant difference was observed in sleep time. Therefore, it is possible to see the difference in whether the time during the day when the subject could be active is spent in a static or resting state, such as sitting or lying down, or whether the time is spent engaging in some kind of physical activity. Furthermore, previous studies [23,24] have shown that even if the amount of physical activity in leisure time increased, the effect of sitting time on mortality risk reduction was small. Therefore, sitting and lying time is important even if physical activity time is high. In order to understand physical activity status, it may be better to prioritize asking the amount of time spent sitting or lying down during the day rather than the amount of activity.

In this study, two types of rhythm-based walking imagery were assessed: mental C-walking rhythm, which is an image of C-walking, and mental M-walking rhythm, which is an image of M-walking. In addition, the ∆ mental C-walking rhythm and ∆ mental M-walking rhythm, which are the errors from the actual walking rhythm, were also calculated. Furthermore, the ability to tap accurately to the imagined walking rhythm was assessed as visual rhythm, while potential rhythmic ability was assessed as auditory rhythm. The walking video used to measure visual rhythm was 100 BPM, and although there was a significant difference between groups, the subjects were able to tap at approximately 100 BPM, indicating that they had the ability to tap almost accurately in accordance with each imagined walking. In auditory rhythm, the HA-Group had a lower ∆ auditory rhythm than the LA-Group, so it is likely that they had the rhythmic ability to tap in time with the rhythm of music. This suggests that the HA-Group potentially had a higher ability to accurately detect rhythms, but it is difficult to clarify the reason at this time. Previous studies have reported that the cerebellum and basal ganglia are involved in rhythmic ability [25,26], and that the cerebellum increases perceptual sensitivity by directing attention [26]. It was also stated that this function in the cerebellum exists for rhythm internalization and predictive motor control [25,27,28]. In other words, if the rhythm is correctly generated in the brain, by reproducing and evaluating that rhythm, it may be possible to predictably evaluate the exercise that will be performed in the future. In this study, the HA-Group had lower MoCA-J scores and seemed to have lower cognitive function than the LA-Group, but they were also older. ANCOVA with age as an adjustment factor revealed no difference in MoCA-J score between the two groups. In addition, significant differences in visual rhythm, auditory rhythm, and mental C-walking rhythm were also found to be significantly influenced by age. In addition to age, it is also necessary to consider the influence of latent rhythmic ability, so similar results were obtained in an ANCOVA in which ∆visual rhythm and ∆auditory rhythm regarding visual and auditory rhythms were also used as adjustment factors. Regarding ∆ mental M-walking rhythm, the HA-Group had a significantly lower value than the LA-Group. Therefore, the evaluation of motor imagery based on rhythm is an evaluation for predicting movement in advance, and it seems useful to use the mental maximum walking rhythm. Furthermore, by performing this evaluation, it may be possible to predict the amount of physical activity of the subject.

The reason why there was no significant difference in mental normal walking rhythm is that both groups walk approximately 40–60 min hours a day, and this may be related to the fact that the walking time was about the same. M-walking is not actively performed in daily life, but the fact that the HA-Group’s ∆M-walking rhythm was low suggests that it may be performed at some point in daily life. This may have been reproduced as a motor imagery of walking rhythm. In the future, it will be necessary to evaluate the situations and frequency of M-walking in daily life.

As a limitation of this study, the sample size was initially small, so it is necessary to expand the sample size in the future to collect more accurate data. Additionally, since a prior estimation of the sample size was not conducted in this study, it is necessary for future research to perform a prior sample size estimation and determine the sample size based on power analysis. Furthermore, conducting studies with different target groups and under various conditions to confirm the reproducibility of the results would be desirable. Second, it was not possible to clarify the difference between those who had latent rhythmic ability and those who did not. Although this study performed an analysis using ANCOVA with adjustment factors, it was not possible to accurately select subjects using the definition. In the future, it will be necessary to investigate what influences the differences in rhythm ability between people. Thirdly, previous studies [29] have shown that the environment in which measurements are taken can also influences the motor imagery of walking. Therefore, future research should consider the specific environment in which measurements are conducted. Finally, since this was a cross-sectional study, we were able to show that the image of gait rhythm and the amount of time spent sitting or lying down during the day were involved, but the causal relationship is unclear. Therefore, by setting up a research period, we would like to clarify the causal relationship between changes in motor imagery and changes in the amount of physical activity in the subjects’ walking rhythm. We believe that by continuing to investigate these matters, we will be able to further clarify the relationship between physical activity and motor imagery, which will be useful in the evaluation and approach of physical therapy.

The method of evaluating mental M-walking rhythm used in this study may have the potential to easily and objectively identify individuals who may have reduced physical activity. Additionally, by applying this assessment method, rhythm-based motor imagery training can be recommended, which could contribute to promoting physical activity in individuals with reduced activity levels as well as aiding cognitive function training.

## 5. Conclusions

This study revealed that there is a relationship between physical activity and the error between one’s own walking rhythm image and the actual walking rhythm. iTUG, which imagines the time required, is useful for assessing motor imagery, but further assessment of motor imagery using rhythm may also be useful. Furthermore, people with a large difference between their own maximum walking rhythm and their actual M-walking rhythm may be those who spend a lot of time sitting a day and have little physical activity.

## Figures and Tables

**Table 1:** Characteristics of participants.

Variables	Mean (SD) or Number (%)							
	Total		HA-Group		LA-Group		*p* Value	Effect Size
	n = 28		n = 8		n = 20			(*r*, *φ*)
Age, y	44.6	(27.1)	65.1	(18.9)	36.6	(25.8)	0.009	0.48
Sex, male	4	(14.3)	1	(12.5)	3	(15.0)	n.s.	
Height, cm	158.9	(7.9)	155.4	(6.5)	160.3	(8.2)	n.s.	0.28
Weight, kg	52.9	(6.5)	51.6	(5.2)	53.5	(6.9)	n.s.	0.13
BMI, kg/m^2^	20.9	(1.9)	21.4	(1.6)	20.8	(2.1)	n.s.	0.14
Sitting or lying down time, h/day	6.2	(4.1)	2.1	(1.2)	7.9	(3.6)	0.0001	0.66
Sleep time, h/day	6.6	(1.4)	6.5	(1.3)	6.6	(1.5)	n.s.	0.03

Abbreviations: HA-Group, high activity group; LA-Group, low activity group; BMI, Body Mass Index; n.s., not significant. Student’s *t*-test.

**Table 2:** Comparison between two groups of HA-Group and LA-Group.

Crude Model		Mean (SD) or Number (%)							
Variables		Total		HA-Group		LA-Group		*p* Value	Effect Size
		n = 28		n = 8		n = 20			(*r*, *φ*)
MoCA-J, score		26.5	(2.9)	24.1	(3.2)	27.5	(2.2)	0.004 ^a^	0.53
Rhythm ability, BPM									
	Visual rhythm	99.8	(2.6)	98.3	(2.2)	100.4	(2.7)	0.049 ^b^	0.35
	Auditory rhythm	145.9	(54.8)	178.4	(48.2)	130.7	(53.9)	0.023 ^b^	0.41
	Mental C-walking rhythm	88.6	(22.1)	104.3	(24.0)	82.3	(190.0)	0.017 ^a^	0.45
	Mental M-walking rhythm	124.5	(36.9)	156.7	(42.0)	111.6	(27.5)	0.002 ^a^	0.55
Actual C-walking rhythm, BPM		118.6	(10.2)	121.8	(11.4)	117.3	(9.9)	n.s.	0.20
Actual M-walking rhythm, BPM		153.0	(22.1)	139.1	(8.6)	158.6	(24.1)	0.025 ^b^	0.39
Δ Rhythm ability, %									
	Δ Visual rhythm	0.2	(2.6)	1.8	(2.2)	−0.4	(2.6)	0.049 ^b^	0.35
	Δ Auditory rhythm	20.1	(38.4)	−2.2	(31.5)	31.4	(38.5)	0.023 ^b^	0.41
	Δ Mental C-walking rhythm	−14.9	(31.6)	−37.6	(28.8)	−5.8	(30.3)	0.015 ^a^	0.46
	Δ Mental M-walking rhythm	23.1	(31.9)	−8.9	(23.9)	35.9	(26.2)	0.001 ^a^	0.63
Walking time per week, h		6.7	(7.9)	4.9	(6.9)	7.5	(8.3)	n.s.	0.22
TUG, s		8.2	(1.7)	8.9	(2.7)	7.9	(1.0)	n.s.	0.30
iTUG, s		6.5	(1.7)	6.1	(2.0)	6.6	(1.5)	n.s.	0.14
Δ TUG, %		23.9	(27.8)	37.1	(39.6)	18.6	(20.5)	n.s.	0.29

Abbreviations: HA-Group, high activity group; LA-Group, low activity group; MoCA-J, Japanese version of Montreal Cognitive Assessment; C-walking, comfortable walking; M-walking, maximum walking; TUG, Timed Up and Go test; iTUG, imaged Timed Up and Go test; n.s., not significant; ^a^, Student’s *t*-test; ^b^, Mann–Whitney U test;

**Table 3:** Comparison between two groups of HA-Group and LA-Group adjusted for age.

Adjusted Model 1		Mean (SD) or Number (%)							
Variables		Total		HA-Group		LA-Group		*p* Value	Effect Size
		n = 28		n = 8		n = 20			(*η*^2^)
MoCA-J, score		26.5	(2.9)	24.1	(3.2)	27.5	(2.2)	n.s.	0.12
Rhythm ability, BPM									
	Visual rhythm	99.8	(2.6)	98.3	(2.2)	100.4	(2.7)	n.s.	0.12
	Auditory rhythm	145.9	(54.8)	178.4	(48.2)	130.7	(53.9)	n.s.	0.11
	Mental C-walking rhythm	88.6	(22.1)	104.3	(24.0)	82.3	(19.0)	n.s.	0.07
	Mental M-walking rhythm	124.5	(36.9)	156.7	(42.0)	111.6	(27.5)	0.031	0.17
Actual C-walking rhythm, BPM		118.6	(10.2)	121.8	(11.4)	117.3	(9.9)	n.s.	0.00
Actual M-walking rhythm, BPM		153.0	(22.1)	139.1	(8.6)	158.6	(24.1)	n.s.	0.05
Δ Rhythm ability, %									
	Δ Visual rhythm	0.2	(2.6)	1.8	(2.2)	−0.4	(2.6)	n.s.	0.12
	Δ Auditory rhythm	20.1	(38.4)	−2.2	(31.5)	31.4	(38.5)	n.s.	0.12
	Δ Mental C-walking rhythm	−14.9	(31.6)	−37.6	(28.8)	−5.8	(30.3)	n.s.	0.09
	Δ Mental M-walking rhythm	23.1	(31.9)	−8.9	(23.9)	35.9	(26.2)	0.009	0.24
Walking time per week, h		6.7	(7.9)	4.9	(6.9)	7.5	(8.3)	n.s.	0.001
TUG, s		8.2	(1.7)	8.9	(2.7)	7.9	(1.0)	n.s.	0.03
iTUG, s		6.5	(1.7)	6.1	(2.0)	6.6	(1.5)	n.s.	0.052
Δ TUG, %		23.9	(27.8)	37.1	(39.6)	18.6	(20.5)	n.s.	0.08

Abbreviations: HA-Group, high activity group; LA-Group, low activity group; MoCA-J, Japanese version of Montreal Cognitive Assessment; C-walking, comfortable walking; M-walking, maximum walking; TUG, Timed Up and Go test; iTUG, imaged Timed Up and Go test; n.s., not significant; comparisons were made using analysis of covariance (ANCOVA).

**Table 4:** Comparison between two groups of HA-Group and LA-Group adjusted for age, Δ visual rhythm, and Δ auditory rhythm.

Adjusted Model 2		Mean (SD) or Number (%)							
Variables		Total		HA-Group		LA-Group		*p* Value	Effect Size
		n = 28		n = 8		n = 20			(*η*^2^)
MoCA-J, score		26.5	(2.9)	24.1	(3.2)	27.5	(2.2)	n.s.	0.12
Rhythm ability, BPM									
	Visual rhythm	99.8	(2.6)	98.3	(2.2)	100.4	(2.7)	n.s.	0.12
	Auditory rhythm	145.9	(54.8)	178.4	(48.2)	130.7	(53.9)	n.s.	0.11
	Mental C-walking rhythm	88.6	(22.1)	104.3	(24.0)	82.3	(19.0)	n.s.	0.07
	Mental M-walking rhythm	124.5	(36.9)	156.7	(42.0)	111.6	(27.5)	0.019	0.24
Actual C-walking rhythm, BPM		118.6	(10.2)	121.8	(11.4)	117.3	(9.9)	n.s.	0.00
Actual M-walking rhythm, BPM		153.0	(22.1)	139.1	(8.6)	158.6	(24.1)	n.s.	0.05
Δ Rhythm ability, %									
	Δ Visual rhythm	0.2	(2.6)	1.8	(2.2)	−0.4	(2.6)	n.s.	0.12
	Δ Auditory rhythm	20.1	(38.4)	−2.2	(31.5)	31.4	(38.5)	n.s.	0.12
	Δ Mental C-walking rhythm	−14.9	(31.6)	−37.6	(28.8)	−5.8	(30.3)	n.s.	0.09
	Δ Mental M-walking rhythm	23.1	(31.9)	−8.9	(23.9)	35.9	(26.2)	0.008	0.30
Walking time per week, h		6.7	(7.9)	4.9	(6.9)	7.5	(8.3)	n.s.	0.001
TUG, s		8.2	(1.7)	8.9	(2.7)	7.9	(1.0)	n.s.	0.03
iTUG, s		6.5	(1.7)	6.1	(2.0)	6.6	(1.5)	n.s.	0.052
Δ TUG, %		23.9	(27.8)	37.1	(39.6)	18.6	(20.5)	n.s.	0.08

Abbreviations: HA-group, high activity group; LA-Group, low activity group; MoCA-J, Japanese version of Montreal Cognitive Assessment; C-walking, comfortable walking; M-walking, maximum walking; TUG, Timed Up and Go test; iTUG, imaged Timed Up and Go test; n.s., not significant; comparisons were made using analysis of covariance (ANCOVA).

## Data Availability

Data is contained within the article.

## Abbreviations

The following abbreviations are used in this manuscript:

HA-Group	High activity group
LA-Group	Low activity group
MoCA-J	Japanese version of Montreal Cognitive Assessment
C-walking	Comfortable walking
M-walking	Maximum walking
TUG	Timed Up and Go test
iTUG	imaged Timed Up and Go test
